# What to expect when you want to be expecting: time-to-pregnancy expectations and anticipated worry and help-seeking among a nationally representative survey of women in Uganda

**DOI:** 10.1186/s12978-025-02148-1

**Published:** 2025-11-26

**Authors:** Suzanne O. Bell, Fredrick Makumbi, Haley L. Thomas, Simon P.S. Kibira, Caroline Moreau, Linnea Zimmerman

**Affiliations:** 1 Department of Population, Family and Reproductive Health, Johns Hopkins Bloomberg School of Public Health, Baltimore, Maryland USA; 2 Department of Community Health and Behavioral Sciences, School of Public Health, Makerere University, Kampala, Uganda; 3 Soins Primaires et Prevention, CESP Centre for Research in Epidemiology and Population Health, U1018, Inserm, Villejuif, France; 4 Department of Epidemiology and Biostatistics, School of Public Health, Makerere University, Kampala, Uganda

**Keywords:** Infertility, Uganda, Cross-sectional, Survey

## Abstract

**Background:**

It is important to understand time-to-pregnancy expectations and when women begin to worry or seek help for fertility-related concerns, particularly in sub-Saharan Africa, where infertility is highly stigmatized and there is considerable pressure to conceive quickly. Yet our understanding of this topic is informed by a small body of literature often involving selective clinic-based samples of those receiving infertility care. This study aims to better understand Ugandan women’s expected time-to-pregnancy and their anticipated emotional and behavioral responses to time spent trying as well as its alignment with the 12-month clinical threshold for infertility.

**Methods:**

We use population-based cross-sectional data of women aged 15–49 in Uganda (*n* = 4227), limiting our analytic sample to women who had ever had sex (*n* = 3741). Our outcomes of interest were time-to-pregnancy expectations and related worry and help-seeking. We operationalized time-to-pregnancy expectations as a continuous measure, in months, and worry and help-seeking as binary measures, with a 12-month and a 12- and 24-month cut-off, respectively. We fit multivariable Tobit and logistic regression models to identify sociodemographic factors associated with time-to-pregnancy expectations and related worry and help-seeking.

**Results:**

Half of women think it typically takes less than one month to conceive. The majority anticipated that they would begin to worry (82.4%) and seek help (77.3%) before reaching the 12-month clinical threshold for infertility. Women with children, with perceived difficulties conceiving, and with longer time-to-pregnancy expectations had decreased odds of worrying or seeking help before 12 months. Over 10% of women anticipated that they would seek help after 24 months of trying, with those who had longer time-to-pregnancy expectations having increased odds of seeking help after 24 months of trying. Time-to-pregnancy expectations, worry, and help-seeking were highly normative, with 27–36% of the variability in these outcomes explained by the woman’s geographic community.

**Conclusion:**

Our study suggests that the clinical threshold for infertility may not align with individuals’ expectations and concerns related to delayed childbearing in Uganda. More education about conception and suggested care-seeking timelines, along with early psychosocial support, could help women navigate fertility concerns, especially in cultures where delayed pregnancy leads to stigma and social consequences.

## Abstract (plain language summary)

It is important to understand time-to-pregnancy expectations and when women begin to worry or seek help for fertility-related concerns, particularly in sub-Saharan Africa, where infertility is highly stigmatized and there is considerable pressure to conceive quickly. This study uses population-based data to examine women’s expectations about time-to-pregnancy and when they would begin to worry or seek help. We use data from 3,741 sexually active women in Uganda to better understand expected time-to-pregnancy and when women would begin to worry and seek help in response to duration trying to conceive. Half of the women in our sample believe conception typically happens in less than a month. Most women expected to start worrying (82.4%) and seek help (77.3%) before reaching the clinical definition of infertility (12 months of trying). However, women with children, those who believed they had difficulty conceiving, and those who expected a longer time-to-pregnancy were less likely to worry or seek help before 12 months. Over 10% of women would wait more than 24 months before seeking help, much longer than recommended. Women with longer time-to-pregnancy expectations were more likely to wait longer than 24 months. Community norms strongly shaped time-to-pregnancy expectations, worries, and help-seeking behaviors. Our study suggests the clinical threshold for infertility may not align with individuals’ expectations and concerns related to delayed childbearing in Uganda. More education about conception and suggested help-seeking timelines, along with early psychosocial support, could help women navigate fertility concerns, especially in cultures where delayed pregnancy leads to stigma and social consequences.

## Background

Infertility – defined clinically as the inability to conceive within 12 months of regular unprotected heterosexual intercourse – affects an estimated 13% (95% CI 11–15) of people worldwide [1]. People suffering from infertility may experience a range of negative impacts on their psychosocial well-being, marriage, and finances [2–7]. Additionally, longer time-to-pregnancy for those who ultimately conceive may indicate underlying health issues that can lead to adverse pregnancy outcomes and shorter life expectancy [8–12].

Sub-Saharan Africa, which is part of the region (Africa) with the highest estimated levels of infertility globally at 16% (95% CI 10–26) [1], has considerable familial and societal pressure to conceive [13–15]. This is especially true for women, as their transition to adulthood is often contingent on having children, and parenthood brings respect within the home and broader community [13, 16, 17]. The patrilineal marriage systems and bride price present in much of Africa puts particular pressure on women to conceive shortly after marriage [13, 18]. This may contribute to observed fears of infertility, whereby people have anticipatory concerns about their ability to conceive [14, 19]. It may also inform people’s expectations of how long it should take to conceive, particularly if coupled with blame for delayed conception [4, 15, 20, 21]. These expectations may lead people to self-identify as having a fertility problem despite not yet reaching the 12-month clinical threshold [22, 23]. In contrast, others may not identify themselves as having an issue well after the 12-month threshold of trying in order to avoid the stigma (including internalized) associated with the label [14] or due to lack of knowledge regarding when to seek care for difficulties conceiving [24].

Existing literature suggests that many factors, including gender, education, parity, fertility preferences, duration of pregnancy attempt, community fertility expectations, and broader cultural context, influence perceptions of having a fertility problem [18, 25–27]. Self-identification of a fertility problem is a prerequisite for infertility-related responses such as seeking formal or informal help (i.e., at a hospital or clinic, from a religious leader, etc.) [28, 29]. Yet, our understanding of who and when people in sub-Saharan Africa might seek help for difficulties conceiving is informed by a small body of literature that includes few quantitative studies, typically relying on clinic-based samples of people who are receiving infertility treatment, thus capturing a highly selective portion of those who may need infertility-related care [25, 26, 30–35]. Additionally, these studies provide no information that relates the timing of help-seeking to expectations regarding time to conception or concerns about fertility problems. To our knowledge only one study has directly assessed perceptions of primary infertility, with less than 1 in 5 (18%) respondents in this study in one state of Nigeria indicating a couple who had not conceived within one year of marriage would be considered infertile [36]. This suggests the vast majority of people’s perceptions of infertility do not align with the clinically defined 12-month timeframe. Thus it is unclear how relevant the clinical timeframe for diagnosing infertility – the focus of much of the public health literature on this topic [1, 37] – is in sub-Saharan Africa. Furthermore, despite qualitative evidence of the role of the community in shaping the timeframe within which people are expected to conceive [18], the community’s influence on time-to-pregnancy expectations and help-seeking has not been quantitatively examined.

This study aims to better understand women’s expected time-to-pregnancy and their anticipated emotional and behavioral responses to time spent trying to become pregnant. We examine women’s expectations and anticipated responses in relation to the 12-month threshold, as this is the clinical definition that the global community has coalesced around in recent years and is used for identifying those eligible for treatment [37]. Using this cutoff allows us to assess potential mismatches between perceived fertility issues and clinically defined infertility, as well as early versus delayed utilization of fertility care. Specifically, we aim to (1) estimate how long women think it typically takes to become pregnant, when they would begin to worry about their ability to become pregnant, and when they would seek help for difficulties conceiving; and (2) identify which women are more likely to worry and/or seek help for difficulties getting pregnant prior to reaching the 12-month clinical threshold and which women are more likely to delay help-seeking more than 24 months after the onset of trying, well beyond the clinical threshold at which women become eligible for biomedical infertility-related care. In relation to both aims, we also seek to determine the normative nature of these time-to-pregnancy expectations and responses by estimating the extent to which the community where one lives influences these outcomes.

To achieve these aims, we use data from a nationally representative population-based survey of reproductive-aged women in Uganda, addressing the limitation of existing related research that relies on clinic-based, small, and/or qualitative samples. Uganda is a high fertility country in East Africa; women have on average 4.6 children over their reproductive lives [38]. However, desired fertility is slightly higher at 4.8, with an estimated 15% of women trying to conceive experiencing infertility (irrespective of parity) [39] and nearly half (46%) of women at the end of their reproductive years (aged 44–48) having fewer children than desired [40]. There is considerable stigma surrounding infertility in this region, which puts pressure on women to conceive quickly [13, 17, 41]. Yet, evidence suggests less than one-third of facilities in Uganda offer any infertility-related services, likely far fewer of which offer advanced treatments [42–44]. Having additional insight into people’s expectations around conception in this context will identify the extent to which earlier intervention to reduce stigma and ensure people’s expectations align with likely time-to-pregnancy are needed.

## Methods

### Data

Data for this study come from Performance Monitoring for Action (PMA), a longitudinal survey research project that administers nationally or sub-nationally representative surveys in eight countries in sub-Saharan Africa and Asia to monitor women’s sexual and reproductive health indicators. Surveys are administered annually by trained female interviewers using smartphones with the Open Data Kit (ODK) application.

We specifically used PMA Uganda data. In Phase 1, which occurred between September and October of 2020, PMA Uganda employed a multi-stage cluster design with urban-rural strata and probability proportional to size sampling of 122 geographic areas (clusters) to obtain a nationally representative sample of 4,023 households (97.0% response rate), including 3,938 females aged 15–49 (96.8% response rate). The study followed households and women from the Phase 1 data collection as well as a random sample of replacement households to account for loss to follow-up, enabling continued calculation of nationally representative estimates in subsequent phases. The Phase 3 data, which we used in this study, included an additional 19 clusters (141 total) and were collected between September and October of 2022. The final sample included 4,430 households (96.4% response rate) and 4,227 females aged 15–49 (96.4% response rate). Household and female surveys were administered face-to-face in English or a local language (Luganda, Ngakarimojong, Runyankole-Rukiga, Runyoro-Rutooro, Luo, Lugbara, Ateso, and Lusoga).

All adult female respondents and emancipated minors provided written informed consent to participate while girls aged 15–17 provided assent and a parent provided written consent. The Johns Hopkins University Bloomberg School of Public Health institutional review board and the Makerere University School of Public Health research and ethics committee provided ethical approval for the study protocol. The protocol was also registered with the Uganda National Council for Science and Technology. Additional details about the PMA project can be found at pmadata.org.

### Measures

The PMA female survey includes questions related to sociodemographic characteristics, contraception, sex, relationships, fertility, and fertility preferences. The Phase 3 survey included additional questions regarding respondent’s time-to-pregnancy expectations and their responses to longer-than-expected time-to-pregnancy, which were assessed via three survey items: 1) How long does it typically take for a woman to become pregnant?; 2) If you were trying to become pregnant, after how long would you start worrying that you could not become pregnant?; 3) How long after you started trying to become pregnant would you wait before seeking help? Interviewers recorded responses in units of days, weeks, months, or years, specifying the corresponding value. All responses were converted to months and explored continuously and categorically (< 1 month, 1–2 months, 3–6 months, 7–12 months, 13–24 months, > 24 months, Don’t know/no response). We also created binary variables identifying women who would begin to worry or seek help prior to the clinical infertility definition (< 12 months = 1, ≥12 months = 0). For help-seeking, we also created a binary variable to indicate potential delayed help-seeking (≥24 months = 1, < 24 months = 0), well past the recommended timeframe for seeking infertility-related clinical care once eligible at 12 months.

We examined differences in time-to-pregnancy expectations, time-to-pregnancy worry, and time-to-pregnancy help-seeking for infertility across several sociodemographic characteristics, including age [15–49], education (none, primary, secondary or higher), marital status (currently married/cohabiting, divorced or separated/widowed, never married), wealth tertiles generated via principal component analysis based on household assets, building materials, and water and sanitation sources, and residence (urban, rural). We also assessed whether time-to-pregnancy expectations, worry, and help-seeking varied by reproductive characteristics, which included parity (0, 1, 2–3, 4+), current use of any contraception (no, yes), respondent perceived fecundity and partner perceived fecundity (think can have children, do not know if can have children, has difficulties having children, sure can not have children [anymore], can have children but doctor has recommended against it [respondent only]), fertility preferences (wants or prefers no more children, wants more children, undecided, says she can’t get pregnant), and currently trying to become pregnant (no, yes).

### Analysis

We limited our analytic sample to those who had ever had sex as we believe questions about fertility problems were more salient and easier to answer for this group, as suggested by the higher nonresponse for those who had never had sex (31.6%−41.5% nonresponse across the three questions). We first examined the distribution of female respondents by their sociodemographic and reproductive characteristics. We then assessed the median and percent distribution of time-to-pregnancy expectations, worry, and help-seeking. Next, we calculated mean time-to-pregnancy expectations by sociodemographic characteristics, assessing statistically significant differences via separate linear regressions for each characteristic. We then identified respondents who would worry before 12 months of trying, would seek help before 12 months of trying, and would seek help after 24 months of trying to get pregnant, and examined the distribution of their sociodemographic characteristics. To determine statistically significant differences for these categorical variables, we used a design-based F-test.

To identify factors independently associated with each of our three outcomes, we conducted multivariable regression. The data are not normally distributed, violating linear regression assumptions. We therefore fit Tobit regression models to assess the relationship between women’s characteristics and the continuous versions of our outcomes, which can better handle data of this nature [45]. We then fit logistic regression models to assess the association between women’s characteristics and their time to worry and help-seeking in relation to the clinical infertility definition (< 12 months, $\:\ge\:$12 months), and for delayed help-seeking ($\:\ge\:$24 months). All models adjusted for education, wealth, parity, residence, respondent’s perceived difficulties conceiving, and fertility preferences. Parity, age, and marital status were collinear; given age and parity are both theoretically relevant for this analysis, we fit adjusted models with age (no parity), parity (no age), and age and parity, and selected the model of best fit (parity only) using Akaike Information Criterion and Bayesian Information Criterion. For each model, we included random effects for the geographic cluster to examine the role of the community in explaining the variability in the outcomes, above and beyond the explanatory power of our covariates. We also ran fixed effects models and used the Hausman test to determine which model was appropriate.

All analyses were conducted in Stata version 16 and included survey weights to account for the complex sampling design, including probability of selection and nonresponse, and to adjust appropriately for geographic clustering. We determined significance using a cutoff of *p* < 0.05.

## Results

In Table 1 we present sample characteristics. Most of the 3,741 female respondents were 20–29 years old (41.0%), had a primary level education (54.6%), were currently married/cohabiting (68.0%), and lived in rural localities (69.0%). More than half had two or more children (65.8%), wanted more children (68.0%), and were not using contraception at the time of the survey (53.3%). The vast majority thought they (88.3%) and their partner (96.0%, among those currently partnered) could have children. Only 10.7% were trying to become pregnant at the time of the survey.

**Table 1:** Characteristics of women aged 15-49 who have ever had sex, Uganda 2022 (*N*=3741)*

		%	*n*	
Age				
15–19		12.3	454	
20–29		41.0	1500	
30–39		30.3	1136	
40–49		16.5	651	
Education				
None		4.5	226	
Primary		54.6	2193	
Secondary		30.6	1027	
Higher		10.3	294	
Marital status				
Currently married/cohabiting		68.0	2554	
Divorced or separated/widowed		17.5	708	
Never married		14.5	478	
Wealth tertile				
Poorest		31.3	1396	
Middle wealthiest		33.2	1283	
Wealthiest		35.4	1062	
Parity				
0		15.2	517	
1		18.9	696	
2–3		25.7	975	
4+		40.1	1552	
Residence				
Rural		69.0	2439	
Urban		31.0	1302	
Currently using contraception				
No		53.3	2027	
Yes		46.7	1714	
Respondent perceived fecundity				
Think can have children		88.3	3280	
Do not know if can have children		3.3	126	
Has difficulties having children		3.6	130	
Sure cannot have children (anymore)		4.4	190	
Can have children but doctor has recommended against it		0.4	14	
Respondent perception of partner fecundity				
Think can have children		96.0	2660	
Has difficulties having children		0.8	21	
Sure cannot have children (anymore)		0.8	28	
Do not know if can have children		2.5	73	
Fertility preferences				
Wants or prefers no more children		27.7	1052	
Wants more children		68.0	2518	
Undecided		2.9	108	
Says she can’t get pregnant		1.4	53	
Currently trying to become pregnant				
No		89.3	2086	
Yes		10.7	270	
Total		100.0	3741	

*N values are unweighted, percentages are weighted; Ns may not add to total due to missingness or skip patterns in the case of respondent perception of partner fecundity (only asked of currently partnered respondents) and whether currently trying to become pregnant (not asked of respondents who say they do not want any or anymore children, who have had a hysterectomy, or who are currently pregnant)

Distributions of the time-to-pregnancy expectations, worry, and help-seeking variables are presented in Table 2. The median time-to-pregnancy expectation was 1 month (interquartile range [IQR] 0–2 months). When assessed categorically, most respondents expected it would typically take less than one month for a woman to become pregnant, with more than three-quarters of all respondents (79.1%) expecting it to take less than 2 months. The median time after which women would start to worry that they could not become pregnant was 6 months (IQR 2–12 months), with the most common response being 7–12 months (25.8%), followed by 3–6 months (23.1%) and 1–2 months (21.6%). More than one in six women (17.6%) would not begin worrying until after 12 months, the clinical definition of infertility, and half of those (8.7%) would not begin to worry until after 24 months. The median reported time to help-seeking after trying to become pregnant was 12 months (IQR 3–12 months). The most common timeframe for help-seeking was 7–12 months (29.1%), though a quarter of women indicated they would seek help within the first 2 months (25.5%).

**Table 2:** Time-to-pregnancy expectations and responses among women aged 15–49 who have ever had sex, Uganda 2022* (*N* = 3741)

		%	*n*
How long does it typically take for a woman to become pregnant?			
Median (IQR)		1 month (0–2 months)	
< 1 month		50.2	1627
1–2 months		28.9	945
3–6 months		12.2	421
7–12 months		5.5	201
13–24 months		2.0	76
> 24 months		1.1	39
If you were trying to become pregnant, after how long would you start worrying that you could not become pregnant?			
Median (IQR)		6 months (2–12 months)	
< 1 month		11.9	420
1–2 months		21.6	715
3–6 months		23.1	766
7–12 months		25.8	927
13–24 months		8.9	340
> 24 months		8.7	325
How long after you started trying to become pregnant would you wait before seeking help?			
Median (IQR)		12 months (3–12 months)	
< 1 month		8.0	303
1–2 months		17.5	568
3–6 months		22.7	735
7–12 months		29.1	1039
13–24 months		12.4	409
> 24 months		10.4	404

Percent (*n*) of don’t know/no response by question: expectations, 12.4% (*n* = 426); worry, 6.7% (*n* = 195); help-seeking, 7.6% (*n* = 222)

Breakdown of don’t know vs. no response by question: expectations, *n* = 422, *n* = 4; worry, *n* = 187, *n* = 8; help-seeking, *n* = 210, *n* = 12.

Those who reported unrealistic values (> 10 years), potentially indicating a misunderstanding of the questions, were excluded from the denominator for their respective questions: expectations, *n* = 6; worry, *n* = 53; help-seeking, *n* = 61

*IQR* interquartile range

*N values are unweighted, percentages are weighted

Mean time-to-pregnancy expectations significantly differed by age, education, parity, and perceived difficulties conceiving (Table 3). Older age was associated with a longer time-to-pregnancy expectation, from 3.1 months (SE 0.8) among those aged 15–19 to 3.5 months (SE 0.6) among those aged 40–49. Greater education was associated with a shorter time-to-pregnancy expectation: those who had no education expected it typically takes 3.7 (SE 1.3) months to conceive, whereas those with higher education thought it would take 1.3 (SE 0.2) months. Those with 1 child had the shortest expected time-to-pregnancy (1.9, SE 0.2), while nulliparous women and those with two or more children anticipated it would take longer to conceive. Having perceived difficulties conceiving was associated with the largest difference in time-to-pregnancy expectations (5.3, SE 0.98 versus 2.6, SE 0.35). In multivariable analysis, women with perceived difficulties conceiving estimated that it would typically take more than three months longer to conceive than those with no perceived difficulties conceiving; no other characteristics were independently related to time-to-pregnancy expectations (Appendix Table A1). Cluster random effects indicate 25% of the variance in time-to-pregnancy expectations is explained by the community in which one lives.

**Table 3:** Mean time-to-pregnancy expectations of women aged 15–49 who have ever had sex in Uganda by background characteristics, 2022 (*N* = 3741)*

	Mean (SE)
Age	
15-19	**3.1 (0.8)**
20-29	**2.1 (0.2)**
30-39	**3.2 (0.5)**
40-49	**3.5 (0.6)**
Education	
None	**3.7 (1.3)**
Primary	**3.2 (0.4)**
Secondary	**2.6 (0.5)**
Higher	**1.3 (0.2)**
Marital status	
Currently married/cohabiting	2.9 (0.3)
Divorced or separated/widowed	2.8 (0.5)
Never married	2.3 (0.6)
Wealth tertile	
Poorest	2.4 (0.3)
Middle wealthiest	3.4 (0.2)
Wealthiest	2.6 (0.5)
Parity	
0	**2.6 (0.6)**
1	**1.9 (0.2)**
2-3	**3.0 (0.4)**
4+	**3.1 (0.5)**
Residence	
Rural	2.9 (0.4)
Urban	2.6 (0.8)
Respondent has difficulties conceiving	
No	**2.6 (0.4)**
Yes	**5.3 (1.0)**
Partner has difficulties conceiving	
No	3.0 (0.4)
Yes	3.8 (1.8)
Fertility preferences	
Wants no more/undecided/says she can't get pregnant	3.0 (0.4)
Wants more	2.7 (0.4)
Total	2.8 (0.4)

*Means are weighted; significant differences between categories of characteristics bolded (*p*-value<0.05)

Results from bivariable analyses of the relationship between characteristics and time-to-pregnancy-related worry and help-seeking are presented in Table 4. Women who would start to worry or seek help before 12 months of trying to become pregnant were significantly more likely to be younger, never married, have no children, have no perceived difficulties conceiving, and want more children. Women who would delay help-seeking until after 24 months of trying to become pregnant were significantly more likely to be older, less educated, married or cohabiting, poorer, have two or more children, live in rural localities, have perceived difficulties conceiving, and report not wanting anymore children/being undecided about more children or saying they can’t get pregnant. In multivariable analyses, women who had two or more children and those who had perceived difficulties conceiving would begin to worry about their ability to conceive approximately two to two-and-a-half months later than those with no children or who had no perceived difficulties, while those residing in urban areas would begin to worry more than three-and-a-half months sooner than rural women (Appendix Table A2). Results for help-seeking were similar. Women's geographic cluster explained roughly one-quarter of the variability in these outcomes.

**Table 4:** Characteristics associated with time-to-pregnancy worry and help-seeking among women aged 15-49 who have ever had sex in Uganda, 2022 (*N* = 3741)*

	Would worry before 12 months of trying		Would seek help before 12 months of trying		Would seek help after 24 months of trying	
	%	*n*	%	*n*	%	*n*
Age						
15-19	**67.2**	**247**	**61.4**	**216**	**8.6**	**38**
20-29	**60.7**	**844**	**52.6**	**733**	**8.3**	**130**
30-39	**55.5**	**582**	**47.2**	**489**	**11.9**	**142**
40-49	**52.9**	**307**	**45.9**	**255**	**14.3**	**94**
Education						
None	52.0	107	39.7	80	**14.7**	**28**
Primary	57.2	1137	50.2	966	**12.1**	**274**
Secondary	60.3	568	54.3	514	**7.5**	**78**
Higher	63.5	168	48.3	133	**8.3**	**24**
Marital status						
Currently married/cohabiting	**55.8**	**1329**	**48.7**	**1133**	**11.6**	**297**
Divorced or separated/widowed	**58.5**	**369**	**50.8**	**319**	**9.3**	**74**
Never married	**72.2**	**282**	**61.7**	**241**	**6.0**	**33**
Wealth tertile						
Poorest	59.8	739	50.8	613	**13.9**	**179**
Middle wealthiest	57.7	690	50.2	583	**9.9**	**127**
Wealthiest	58.0	551	51.3	497	**7.8**	**98**
Parity						
0	**69.2**	**287**	**59.7**	**248**	**5.9**	**33**
1	**63.3**	**390**	**53.5**	**337**	**9.2**	**66**
2-3	**55.8**	**506**	**49.1 **	**445**	**9.4 **	**97**
4+	**54.5**	**797**	**47.6**	**663**	**13.2**	**207**
Residence						
Rural	56.3	1257	48.3	1073	**12.3**	**300**
Urban	63.5	723	56.2	620	**6.2**	**104**
Respondent has difficulties conceiving						
No	**59.2**	**1825**	**51.5**	**1566**	**9.9**	**354**
Yes	**51.1**	**155**	**42.7**	**127**	**16.7**	**50**
Partner has difficulties conceiving						
No	57.0	1436	49.9	1230	11.1	307
Yes	61.0	26	42.9	18	14.4	7
Fertility preferences						
Wants no more/undecided/says she can't get pregnant	**53.9**	**604**	**46.8**	**500**	**12.5**	**156**
Wants more	**60.6**	**1371**	**52.6**	**1188**	**9.5**	**248**
Total	58.5	1980	50.8	1693	10.4	404

*N values are unweighted, percentages are weighted; bolding indicates *p*-value<0.05 based on design-based F-test

We present results from multivariable logistic regressions in Table 5. In relation to the clinical definition of infertility, women with children had decreased odds of worrying before 12 months of trying compared to nulliparous women (aOR_1_ = 0.76, 95% CI: 0.56–1.04; aOR_2−3_=0.61, 95% CI: 0.45–0.83; aOR_4+_=0.63, 95% CI: 0.46−0.86). Additionally, a one month increase in the expected time-to-pregnancy was associated with reduced odds of worrying before 12 months (aOR 0.91, 95% CI: 0.89–0.93). Cluster random effects suggest one’s geographic community explained more than one-quarter (27%) of the variance in time-to-pregnancy worry.

**Table 5:** Bivariable and multivariable logistic regression of characteristics on time-to-pregnancy worry and help-seeking among women aged 15-49 who have ever had sex in Uganda, 2022 (*N*=3741)

	Below 12-month time-to-pregnancy worry		Below 12-month time-to-pregnancy help-seeking		Above 24-month time-to-pregnancy help-seeking	
	OR (95% CI)	aOR (95% CI)	OR (95% CI)	aOR (95% CI)	OR (95% CI)	aOR (95% CI)
Time-to-pregnancy expectations	0.91 (0.89-0.93)^***^	0.91 (0.89-0.93)^***^	0.96 (0.95-0.98)^***^	0.96 (0.95-0.98)^***^	1.04 (1.02-1.05)^***^	1.03 (1.02-1.05)^***^
Education						
None	ref	ref	ref	ref	ref	ref
Primary	1.07 (0.74-1.54)	1.08 (0.73-1.59)	1.33 (0.90-1.96)	1.22 (0.81-1.85)	1.12 (0.65-1.91)	1.32 (0.74-2.36)
Secondary	1.21 (0.82-1.78)	1.11 (0.73-1.69)	1.69 (1.12-2.55)^**^	1.36 (0.87-2.13)	0.73 (0.40-1.30)	1.06 (0.55-2.04)
Higher	1.13 (0.72-1.80)	1.01 (0.60-1.67)	1.24 (0.77-2.02)	0.92 (0.54-1.57)	0.83 (0.40-1.70)	1.30 (0.58-2.91)
Wealth tertile						
Poorest	ref	ref	ref	ref	ref	ref
Middle wealthiest	0.96 (0.78-1.18)	0.97 (0.78-1.22)	0.93 (0.75-1.15)	0.86 (0.68-1.09)	0.67 (0.49-0.91)^**^	0.71 (0.50-0.99)^**^
Wealthiest	0.83 (0.64-1.07)	0.82 (0.61-1.09)	1.03 (0.79-1.35)	0.97 (0.72-1.31)	0.88 (0.60-1.29)	1.01 (0.65-1.57)
Parity						
0	ref	ref	ref	ref	ref	ref
1	0.85 (0.64-1.13)	0.76 (0.56-1.05)*	0.86 (0.65-1.15)	0.77 (0.57-1.06)	1.36 (0.84-2.20)	1.65 (0.94-2.90)*
2-3	0.65 (0.50-0.85)^***^	0.61 (0.45-0.83)^***^	0.70 (0.54-0.92)^***^	0.65 (0.48-0.88)^***^	1.30 (0.83-2.05)^**^	1.37(0.80-2.36)
4+	0.63 (0.49-0.82)^***^	0.63 (0.46-0.86)^***^	0.63 (0.49-0.82)^***^	0.67 (0.49-0.91)^**^	1.97 (1.29-3.02)^***^	1.97 (1.14-3.40)^**^
Residence						
Rural	ref	ref	ref	ref	ref	ref
Urban	1.27 (0.83-1.94)	1.30 (0.84-1.99)	1.24 (0.75-2.03)	1.21 (0.72-2.02)	0.57 (0.33-1.00)^**^	0.58 (0.32-1.05)*
Respondent has difficulties conceiving						
No	ref	ref	ref	ref	ref	ref
Yes	0.67 (0.51-0.87)^***^	0.78 (0.58-1.06)	0.62 (0.46-0.82)^***^	0.68 (0.50-0.94)^**^	1.67 (1.15-2.43)^***^	1.47 (0.96-2.25)*
Fertility preferences						
Wants no more/undecided/says she can't get pregnant	ref	ref	ref	ref	ref	ref
Wants more	1.31 (1.12-1.55)^***^	1.14 (0.93-1.41)	1.35 (1.13-1.60)^***^	1.12 (0.90-1.39)	0.74 (0.58-0.94)^**^	0.98 (0.72-1.35)
Cluster random effects		0.27		0.36		0.35

*p-value<0.1, **p-value<0.05, ***p-value<0.01

In relation to help-seeking before 12 months of trying to conceive, women with two or more children and women with perceived difficulties conceiving similarly had decreased odds of seeking help (aOR_2−3_=0.65, 95% CI 0.48–0.88; aOR_4+_=0.67, 95% CI 0-49-0.91; aOR_difficulties_=0.68, 95% CI: 0.50–0.94, respectively). Longer time-to-pregnancy expectations was again associated with lower likelihood of help-seeking before 12 months of trying (aOR = 0.96, 95% CI: 0.95–0.98), and more than one-third (36%) of the variance in early help-seeking was explained by the cluster.

Several factors were related to potential delays in help-seeking. Women in the middle wealth tertile had decreased odds of seeking help after 24 months compared to the poorest women (aOR = 0.71, 95% CI 0.50–0.99). Women with one child and women with self-perceived difficulties conceiving similarly had marginally (*p* < 0.1) increased odds of waiting until after 24 months before seeking help compared to those with no children and those without self-perceived difficulties conceiving (aOR_1_ = 1.65, 95% CI: 0.94–2.90; aOR_difficulties_=1.47, 95% CI 0.96–2.25, respectively), while women with 4 + children had significantly increased odds of waiting until after 24 months (aOR = 1.97, 95% CI: 1.14–3.40). Urban women had marginally decreased odds of waiting until after 24 months before seeking help compared to rural women (aOR = 0.58, 95% CI: 0.32–1.05). Additionally, each one month increase in expectations about the time it typically takes to conceive was associated with a 3% increase in the odds of waiting to seek help until after 24 months (aOR = 1.03, 95% CI: 1.02–1.05). Lastly, one’s community explained 35% of the variance in seeking help after 24 months.

## Discussion

Our study finds that women in Uganda have shorter expectations for how long it takes women to become pregnant than is expected at a population level, with half anticipating it typically takes less than one month to conceive. Furthermore, 56.6% and 48.2% indicated they would worry and seek help, respectively, within 6 months of trying, well before the 12-month clinical infertility definition. These results highlight a potential tension between perceived fertility issues and clinically defined infertility, with most women in this context growing concerned and seeking help to resolve issues before the clinically defined threshold. This is likely due to the substantial pressure to conceive and repercussions for those who don’t do so quickly after marriage, including negative consequences for one’s relationship, social standing, and mental health [4, 13–15, 18, 22, 27, 46–48]. However, approximately 1 in 10 women would *not* be concerned and would not seek help until after 2 years of trying, signaling a not insubstantial group of women for whom the clinical definition may not resonate.

Nearly half of women think it typically takes less than a month for a woman to conceive, while nearly 8 in 10 women think it typically takes less than two months: this does not align with observed time-to-conception data from other settings. Evidence suggests the probability of conception after a single act of sex without contraception is at most 10% if it occurs close to ovulation but is near 0% in other parts of one’s cycle [49, 50]. Cumulatively, approximately 30% of people conceive in the first month of unprotected intercourse, while just under two-thirds and 80% conceive by 6 and 12 months, respectively, though more recent estimates from Africa suggest these cumulative probabilities may be lower in this context [1, 51, 52]. Women in Nigeria and Côte d’Ivoire appear to have similar expectations regarding the ease of conception as women in our study, with 25% (Nigeria) and 43% (Côte d’Ivoire) believing they would definitely become pregnant after having sex without contraception just once; an additional 30% and 37% thought they would maybe conceive [53]. Low levels of biological reproductive knowledge may contribute to these inaccurate expectations of likely time-to-pregnancy [54].

The expectation of a short time-to-pregnancy may lead many to respond emotionally or act quite soon in a future pregnancy attempt. Most women anticipated that they would begin to worry (82.4%) and seek help (77.3%) before reaching the clinical threshold for infertility (12 months). Women with children, with perceived difficulties conceiving, and with longer time-to-pregnancy expectations had significantly decreased odds of worrying or seeking help before 12 months of trying. These findings highlight the importance of expectations in shaping one’s response to their time trying, as well as the issue of primary versus secondary infertility. Nulliparous women’s earlier anticipated emotional and help-seeking response to resolve difficulties conceiving is consistent with existing research that indicates the psychosocial repercussions of infertility are a greater concern for childless women [5, 20, 55].

Conversely, women with children, with perceived difficulties conceiving, and with longer time-to-pregnancy expectations had significantly increased odds of seeking help after at least 24 months of trying, while urban women and women in the middle wealth tertile had decreased odds of waiting until after 24 months of trying to seek help. For some, this delayed help-seeking may be a result of a reluctance to identify or be identified as someone who is unable to conceive, with existing evidence suggesting that long delays in help-seeking to address difficulties conceiving is in part due to stigma [56]. For others, it appears the 12-month infertility definition is stricter than their own perception of fertility issues and simply does not align with their view of what merits help-seeking [29]. These women may not be concerned about not conceiving quickly – a perception that is likely more common among women with children, as our findings suggest, for whom pressure to conceive is lessened [25, 57] – or do not view conception as something within their control that can or should be intervened upon [58]. Alternatively, perceived geographic or financial inaccessibility of infertility-related services, which are limited in Uganda [41], may have led some women to indicate they would wait longer than actually preferred to seek help given the challenge of accessing care in the formal healthcare system; this may partially explain why women with middle wealth and those residing in urban areas were less likely to report intending to wait more than 24 months compared to their counterparts with less financial resources and those in rural areas. These and other factors have been identified in existing research as influencers of the perception of one having a fertility issue and the related care-seeking choices in sub-Saharan Africa, though this work has typically been among small, highly selective clinic-based samples of women who present for infertility treatment [25–27, 30].

While approximately 90% of women indicated they would seek help for difficulties conceiving within two years of trying, we do not have information on the type of help they would seek. Women in settings across sub-Saharan Africa have reported diverse means to address infertility, from biomedical Western treatment to reliance on herbs, healers, and religious intervention, some of which may actually be harmful [22, 30, 35, 56, 59–61]. As most infertility in this region is secondary and a result of untreated sexually transmitted infections or complications from prior pregnancies, biomedical care is necessary to address these issues [62]. Ensuring adequate availability of services is essential to meeting these couples’ reproductive needs, however, infertility-related care is limited and concentrated in urban settings, creating inequities in access [63, 64].

We found these expectations and anticipated emotional and behavioral responses to be geographically clustered, with 23–36% of the variability in these outcomes explained by the woman’s community (as indicated by the cluster random effects), suggesting these perceptions are highly normative. A qualitative study in Malawi similarly found that individuals’ time-to-pregnancy expectations were heavily influenced by community expectations, resulting in a community-defined “acceptable waiting time until pregnancy” [18]. Individuals perceived themselves as having difficulties conceiving based almost entirely on this normative timeframe, regardless of whether or not there were other indicators of a fertility issue, suggesting that social expectations can heavily influence personal experiences, sometimes overshadowing objective medical assessments [18].

Our results indicate the need to provide additional information about reproduction and likelihood of conception to shift expectations to be more in line with likely timelines, as well as to potentially improve timelines for conception. Low levels of biological reproductive knowledge related to the timing of one’s ovulation and when to have sex to maximize conception may mean that per-menstrual-cycle odds of conceiving are lower than observed in high-resource settings, where much of the prospective research on time-to-pregnancy has been conducted [1]. Data from Demographic and Health Surveys in several low- and middle-income countries indicate that less than one-quarter of reproductive-aged women correctly identified the “middle of the cycle” as the most fertile time, with more women responding incorrectly that “after the period ends” was the most fertile time [54]. While some may conceive in the first month of trying to become pregnant, as most women in our study believed, most will not. More information about fecundity and likely time-to-conception for those trying to conceive in this population is needed.

Our study is not without limitations. We encountered several responses to our three outcomes of interest that we deemed to be unrealistic. These outliers, or values greater than 120 months (10 years), were excluded from analyses (time-to-pregnancy expectations [*n* = 6], worry [*n* = 53], help-seeking [*n* = 61]). It is unclear if these outliers were a data entry issue or if respondents misunderstood the question and potentially reported the age at which they would begin to worry or seek help for difficulties getting pregnant. There were also a number of respondents who reported “Don’t know” for our three outcomes of interest, which, while a valid response (and a potential indication of low biological literacy), we had to exclude them from our analyses (time-to-pregnancy expectations [*n* = 422], worry [*n* = 187], help-seeking [*n* = 210]). Additionally, our outcome questions assumed an emotional or behavioral response to time spent trying to become pregnant (i.e., After how long would you start to worry? After how long would you seek help?), with no response option for those who would not worry or would not seek help after any period. Given the near universal desire for children and cultural expectations surrounding childbearing, however, it seems unlikely that many would never be concerned [65]. We also didn’t ask respondents about the type of help they would seek. Recent findings highlight the role of non-medical help in this setting [30], potentially indicating that time to biomedical care-seeking specifically may be longer. The question “How long does it typically take for a woman to become pregnant?” is also a general perceptions question, and women’s responses may not necessarily reflect how long they think it would take *them* to become pregnant. We believe, however, that this is less of a concern because it is women’s perceptions that inform whether their time-to-pregnancy experience is outside of the norm and if they should worry or seek help. Lastly, nulliparous women in our data include both those who have not tried to conceive and those who have experienced primary infertility. While we adjust for perceived infertility, there is likely some endogeneity in the relationship between parity and our time-to-pregnancy-related outcomes.

Despite these limitations, our study has several strengths. To our knowledge, this is the first assessment of time-to-pregnancy expectations and related emotional and behavioral responses to time spent trying to become pregnant in sub-Saharan Africa. These unique data allow us to directly examine expectations and responses to time-to-pregnancy in relation to clinically defined infertility and identify those who might begin to experience psychosocial consequences and desire help prior to 12 months. Additionally, we use population-based data and can therefore generalize this study’s findings and implications to all sexually active women of reproductive age in Uganda.

## Conclusions

Our study suggests that the clinical infertility timeframe may not align with many individuals’ and couples’ expectations and concerns related to time-to-pregnancy in Uganda. Women and couples need additional information about conception (timing, likelihood) to shift expectations to be more in line with likely timelines but also require access to psychosocial support and services prior to 12 months to address their needs, particularly for those who begin to experience stigma and other negative repercussions as a result of delayed conception.

## Data Availability

Data are available in the Johns Hopkins Research Data Repository. We used the publicly available Uganda Phase 3 household and female survey data (10.34976/6mkm-a674).

## Appendix Table 1

### Multivariable Tobit regression of characteristics on time-to-pregnancy expectations among women aged 15-49 who have ever had sex, Uganda 2022 (N=3741)

**Table:**

Education	
None	ref
Primary	1.29 (–0.62-3.20)
Secondary	0.17 (–1.91-2.25)
Higher	–0.64 (–3.15-1.87)
Wealth tertile	
Poorest	ref
Middle wealthiest	0.32 (–0.77-1.41)
Wealthiest	0.24 (–1.18-1.67)
Parity	
0	ref
1	–0.10 (–1.59-1.39)
2-3	1.00 (–0.41-2.43)
4+	–0.01 (–1.49-1.48)
Residence	
Rural	ref
Urban	–0.28 (–2.48-1.92)
Respondent has difficulties conceiving	
No	ref
Yes	3.66 (2.22-5.10)^*******^
Fertility preferences	
Wants no more/undecided/says she can't get pregnant	ref
Wants more	0.27 (–0.76-1.30)
Cluster random effects	0.25

Age, marital status, and parity collinear; retained parity in final model

**p*<0.1 ***p*<0.05 ****p*<0.01

## Appendix Table 2

### Multivariable Tobit regression of characteristics on time-to-pregnancy worry and help-seeking among women aged 15-49 who have ever had sex, Uganda 2022 (N=3741)

**Table:**

	If you were trying to become pregnant, after how long would you start worrying that you could not become pregnant?	How long after you started trying to become pregnant would you wait before seeking help?
Education		
None	ref	ref
Primary	0.99 (–1.60-3.58)	2.30 (–0.41-5.00)
Secondary	0.43 (–2.38-3.25)	1.17 (–1.77-4.10)
Higher	2.09 (–1.29-5.47)	3.03 (–0.47-6.54)
Wealth tertile		
Poorest	ref	ref
Middle wealthiest	–0.34 (–1.79-1.11)	–1.23 (–2.73-0.26)
Wealthiest	1.41 (–0.49-3.31)	–0.12 (–2.09-1.86)
Parity		
0	ref	ref
1	1.34 (–0.60-3.28)	1.29 (–0.70-3.27)
2-3	1.85 (0.00-3.71)^******^	1.99 (0.99-3.89)^******^
4+	2.54 (0.60-4.48)^*******^	3.45 (1.45-5.45)^*******^
Residence		
Rural	ref	ref
Urban	–3.53 (–6.66- –0.40)^******^	–3.11 (–6.59-0.37)
Respondent has difficulties conceiving		
No	ref	ref
Yes	2.43 (0.48-4.38)^ ******^	2.99 (0.95-5.00)^*******^
Fertility preferences		
Wants no more/undecided/says she can't get pregnant	ref	ref
Wants more	–0.69 (–2.07-0.68)	–0.28 (–1.70-1.15)
EA random effects	0.23	0.26

Age, marital status, and parity collinear; retained parity in final model

**p*<0.1 ***p*<0.05 ****p*<0.01 *****p*<0.001

## Abbreviations

PMA: Performance Monitoring for Action
ODK: Open Data Kit
IQR: Interquartile range
